# Fully Automated, Quality-Controlled Cardiac Analysis From CMR: Validation and Large-Scale Application to Characterize Cardiac Function

**DOI:** 10.1016/j.jcmg.2019.05.030

**Published:** 2019-07-11

**Authors:** Bram Ruijsink, Esther Puyol-Antón, Ilkay Oksuz, Matthew Sinclair, Wenjia Bai, Julia A. Schnabel, Reza Razavi, Andrew P. King

**Affiliations:** a School of Biomedical Engineering and Imaging Sciences, King’s College London, London, United Kingdom; b Department of Adult and Paediatric Cardiology, Guy’s and St Thomas’ NHS Foundation Trust, London, London, United Kingdom; c Biomedical Image Analysis Group, Department of Computing, Imperial College London, London, United Kingdom; d Department of Medicine, Imperial College London, London, United Kingdom

**Keywords:** cardiac aging, cardiac function, cardiac magnetic resonance, CMR feature tracking, machine learning, quality control

## Abstract

**Objectives:**

This study sought to develop a fully automated framework for cardiac function analysis from cardiac magnetic resonance (CMR), including comprehensive quality control (QC) algorithms to detect erroneous output.

**Background:**

Analysis of cine CMR imaging using deep learning (DL) algorithms could automate ventricular function assessment. However, variable image quality, variability in phenotypes of disease, and unavoidable weaknesses in training of DL algorithms currently prevent their use in clinical practice.

**Methods:**

The framework consists of a pre-analysis DL image QC, followed by a DL algorithm for biventricular segmentation in long-axis and short-axis views, myocardial feature-tracking (FT), and a post-analysis QC to detect erroneous results. The study validated the framework in healthy subjects and cardiac patients by comparison against manual analysis (n = 100) and evaluation of the QC steps’ ability to detect erroneous results (n = 700). Next, this method was used to obtain reference values for cardiac function metrics from the UK Biobank.

**Results:**

Automated analysis correlated highly with manual analysis for left and right ventricular volumes (all r > 0.95), strain (circumferential r = 0.89, longitudinal r > 0.89), and filling and ejection rates (all r ≥ 0.93). There was no significant bias for cardiac volumes and filling and ejection rates, except for right ventricular end-systolic volume (bias +1.80 ml; p = 0.01). The bias for FT strain was <1.3%. The sensitivity of detection of erroneous output was 95% for volume-derived parameters and 93% for FT strain. Finally, reference values were automatically derived from 2,029 CMR exams in healthy subjects.

**Conclusions:**

The study demonstrates a DL-based framework for automated, quality-controlled characterization of cardiac function from cine CMR, without the need for direct clinician oversight.

Cardiac magnetic resonance (CMR) enables full coverage of the heart using high spatial and temporal resolution, without the constraints of limited acquisition windows or use of ionizing radiation, as with echocardiography or computedtomography (1). Cine CMR has become the gold standard for non-invasive quantification of cardiac volumes and ejection fraction (EF) (1). However, cine CMR images hold significantly more detailed information that allow for quantification of advanced markers of cardiac function such as ventricular shape (2), ejection and filling rates (3), myocardial wall motion, and myocardial strain (ε) (4,5). These parameters have shown to be valuable biomarkers for earlier detection and monitoring of disease (2–5). However, obtaining them is time and labor intensive. Moreover, although largescale studies have provided meaningful reference values and standards for analysis of cardiac volumes and EF (6,7), such studies are absent for the remaining biomarkers. As a result, the use of these advanced markers in clinical practice has so far been limited.

Recent advances in deep learning (DL) algorithms show great promise for the automation of CMR analysis. Convolutional neural networks (CNNs), have achieved previously unmatched accuracy in many image analysis challenges (8). Using CNNs, a wide set of cardiac functional parameters could potentially be obtained automatically from CMR. Several groups have shown that CNNs can provide accurate enddiastolic and end-systolic cardiac segmentations from CMR in preselected images (9–11). Although these results have gained significant attention, the practical implementation of DL algorithms in clinical practice and research is hindered by a lack of appropriate quality control (QC). Variable image quality, image artefacts, and unusual anatomic variations (not seen during training) are unavoidable in clinical imaging, and can result in significant errors if such images are analyzed automatically. Therefore, robust QC measures to detect (potential) erroneous output are a prerequisite to the translation of DL algorithms into clinical practice (12).

We aim to address this issue by developing a pipeline for comprehensive analysis of cardiac function (cardiac volumes, filling and ejection dynamics and myocardial strain) that includes robust QC mechanisms, which allows for automated cine CMR analysis without clinician oversight. Using our pipeline, we provide reference values for a range of automatically derived cardiac metrics that have not previously been reported in large subject cohorts.

## Methods

### Image Analysis Pipeline

The developed image analysis pipeline consists of a DL algorithm for segmentation of short-axis (SAX) and 2- and 4-chamber long-axis (LAX) cine CMR stacks, automated calculation of cardiac functional parameters and 2 QC steps: 1 before the segmentation and analysis steps (QC1) and 1 after (QC2). For an illustration of the pipeline see the Central Illustration and Video 1. Our pipeline is available for further training and use via the corresponding author.

### Step 1: Pre-analysis Image QC (QC1)

All CMR images were screened for the presence of motion artefacts (artefacts due to inconsistent breath-holding, mistriggering or arrhythmias) and erroneous planning of the 4-chamber view using 2 CNNs: a 2-dimensional CNN with a recurrent long short-term memory layer trained to detect motion artefacts and a 2-dimensional CNN trained to detect erroneous planning of the 4-chamber view (CNN_4Ch_). We have previously published a detailed description of the architecture, training, and validation of both algorithms (13,14).

### Step 2: Image Segmentation

After QC1, a 17-layer CNN (CNN_segment_) was used to segment the left ventricle (LV) and right ventricle (RV), including the LV myocardium, in all frames of the cine CMR. This network has been trained using manual segmentations of cine CMR images in 3,975 subjects, consisting of both healthy volunteers as well as patients with a wide variety of cardiac diseases (10).

### Step 3: Parameter Calculation

After segmentation, the SAX and LAX imaging stacks were aligned using an iterative alignment process to correct for different breath-hold positions and motion between the different cine-acquisitions (15). Next, LV and RV volume curves and LV mass (LVM) were calculated. From the volume curves, end-diastolic volume (EDV), end-systolic volume (ESV), stroke volume (SV), EF, peak ejection rate, peak early filling rate, atrial contribution (AC), and peak atrial filling rate were obtained.

Subsequently, CMR feature tracking (FT) was automatically performed on 3 SAX slices, and the 2- and 4-chamber LAX images. We previously published the details of this method (16). Briefly, CMR FT was performed using the Medical Image Registration ToolKit. The end-diastolic LV wall segmentations were used as the region of interest for the FT algorithm. Global circumferential strain (ε^circ^), radial strain (ε^rad^), and longitudinal strain (ε^long^) were computed from the FT results.

### Step 4: Post-Analysis QC (QC2)

In QC2, we first evaluated the orientation of the images, the presence of missing slices, and the coverage of the segmentations over the heart. We automatically compared the aligned LAX and SAX images and segmentations to determine the image plane intersections (e.g., did the LAX images intersect the mitral valve and apex in SAX?), presence of missing slices (e.g., did the SAX stack cover the full length of the LAX segmentation?), and the coverage of segmentations (did LAX segmentation reach a similar level as the SAX segmentation and vice versa?). Next, the output parameters were inspected. If there was a >10% difference between LV and RV SV or a >10% difference between ventricular volumes on the first and last cardiac phase, the exams were flagged. Lastly, we implemented 2 support vector machine (SVM) classification algorithms to detect abnormalities in the obtained volume (SVM_vol_) and strain curves (SVM_strain_). These SVMs were trained using output of the CNN_segment_ and FT algorithm from 500 UK Biobank subjects (300 healthy subjects and 200 subjects with cardiomyopathy). These datasets were classified by an expert CMR cardiologist as right or wrong/unusual on the basis of the shape of the volume and strain curves, as well as the corresponding functional parameters.

All cases detected during the QC steps were flagged for clinician review.

### Pipeline Validation

We validated our method in 2 ways. First, we compared the results obtained to manual analysis by an experienced CMR cardiologist (Validation1) in 50 healthy volunteers and 50 patients with cardiomyopathy. These cases were not previously used during training of the algorithms and were randomly selected after having successfully passed the algorithm’s QC steps. During the manual analysis, ventricular volumes were segmented at each cardiac phase using commercially available CMR analysis software, CVi42 (Version 5.10.1, Circle, Calgary, Alberta, Canada). With the same software, CMR FT was performed to obtain strain values.

Secondly, we evaluated the ability of the full pipeline to detect errors in the analysis (Validation2) in a further 700 cases (500 healthy subjects and 200 patients with cardiomyopathy) randomly selected from the UK Biobank cohort, again excluding cases used during training. An experienced CMR cardiologist, blinded for the pipeline’s verdict, critically reviewed the segmentations, volume and strain curves and parameters obtained in step 3 and classified them as correct or erroneous. This process was facilitated by visually representing the images with segmentations and outcome-parameters for each case in a single panel to ensure apt identification of errors (Supplemental Figure 1, Video 2).

### Obtaining Reference Values

After validation, we utilized the developed pipeline to obtain reference values. Healthy subjects were selected from a total of 9,619 cases in the UK Biobank that underwent CMR (17), excluding all subjects with a history of cardiovascular disease, cardiovascular risk factors, other systemic diseases, those taking medication for any systemic disease, and subjects with a body mass-index >30 kg/m^2^ (see all exclusion criteria in Supplemental Table 1).

### Statistics

#### Validation1

Dice coefficients were calculated to compare the manual and automated segmentations. Bland-Altman analysis and Pearson’s correlations were used to compare the obtained cardiac volumes, filling and ejection rates, and peak global strains to the manual analysis. To verify the significance of the biases, paired *t*-tests versus zero values were applied. Finally, we compared the mean absolute errors of all parameters between healthy subjects and patients with disease using paired *t*-tests.

#### Validation2

Sensitivity (% of manually labelled erroneous output that was correctly detected by the pipeline during QC), specificity (% of output manually labelled as error-free that was not flagged by the pipeline during QC), and balanced accuracy were calculated for the total pipeline’s performance for volume and strain analysis, as well as for each individual parameter.

#### Reference values

Data were stratified by sex, and age by decade (45 to 54, 55 to 64, and 65 to 74 years), and the means and reference ranges (95% prediction intervals) were defined (18). Outliers, defined a priori as values 3 interquartile ranges below the first or above the third quartile, were removed from the analysis. Cardiac volumes were indexed to body surface area using the Dubois and Dubois formula (19). We used linear regression analysis to assess the impact of age on ventricular volumes, filling and ejection dynamics and strains. For all analyses, p values were corrected using Bonferroni correction for multiple comparisons. A p value of <0.05 after correction was considered statistically significant.

## Results

### Validation1

**Figure 1:**
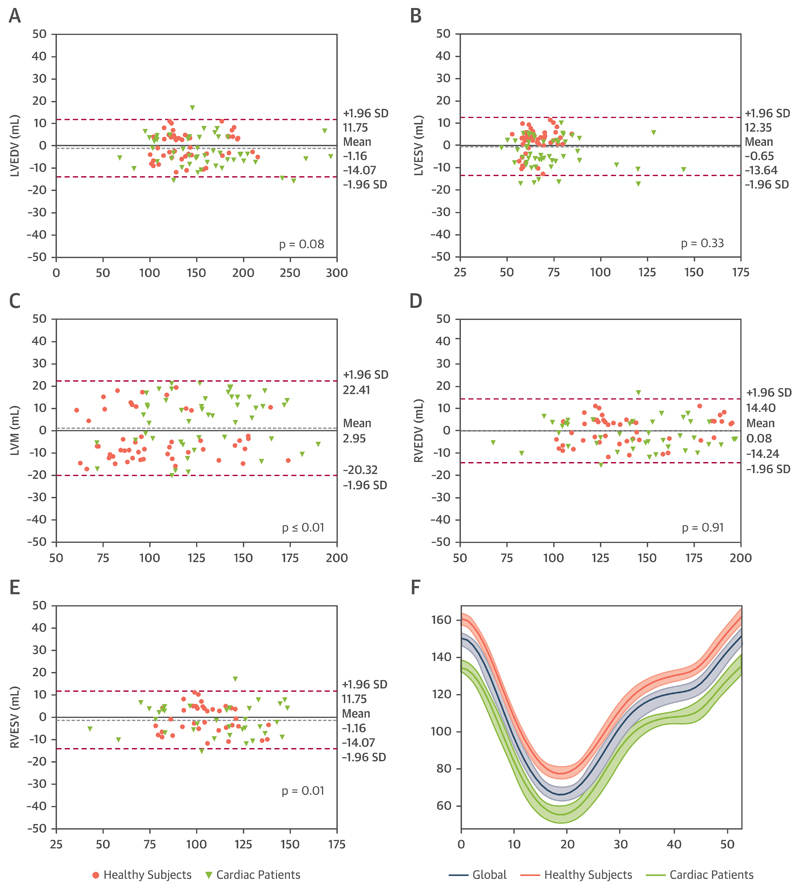
Bland-Altman Plots for Cardiac Volumes **(A)** Left ventricular (LV) end-diastolic volume (LVEDV), **(B)** left ventricular end-systolic volume (LVESV), **(C)** left ventricular end-diastolic mass (LVM), **(D)** right ventricular end-diastolic volume (RVEDV), and **(E)** right ventricular end-systolic volume (RVESV). The **grey dotted line** represents the mean bias; the **pink dotted lines** the limits of agreement. The p values represent the difference in mean bias from zero using a paired t-test. **(F)** The mean error in LV is a normalized volume curve for all cases, and both subgroups is shown.

**Figure 2:**
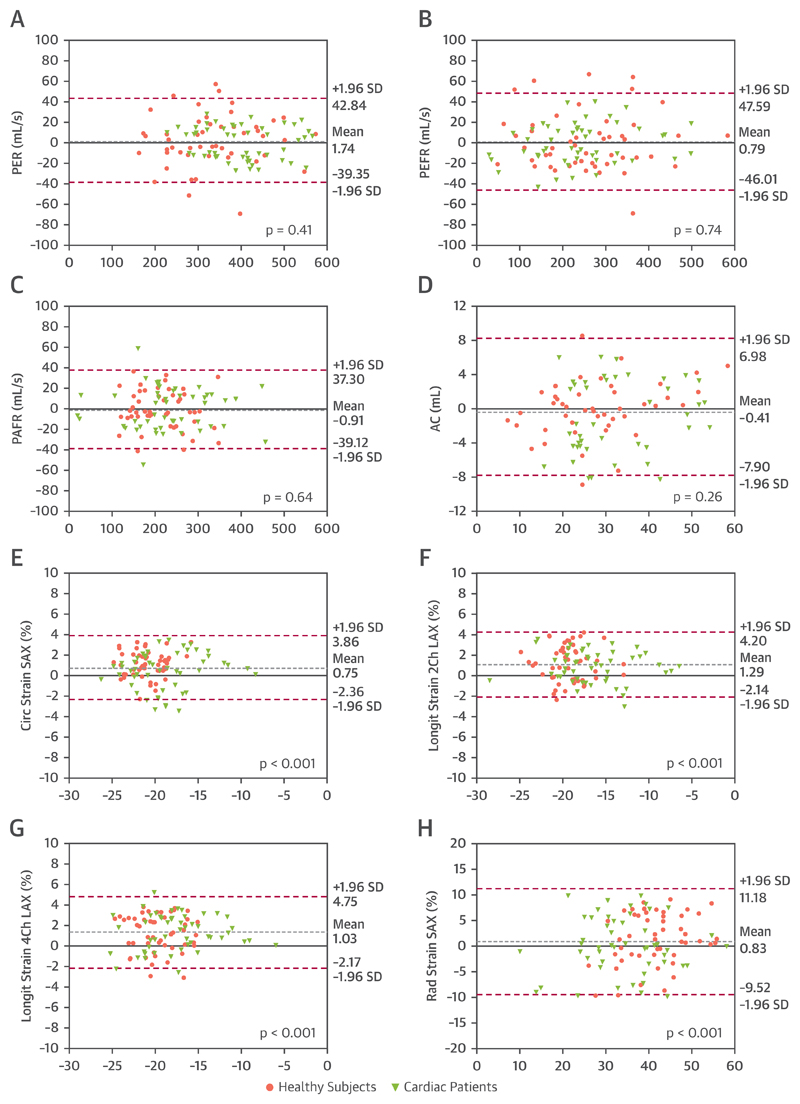
Bland-Altman Plots for LV Filling and Ejection and Global Peak Strain Parameters **(A)** Peak ejection rate (PER), **(B)** peak early filling rate (PEFR), **(C)** peak atrial filling rate (PAFR), **(D)** atrial contribution (AC), **(E)** peak global circumferential strain (Circ), **(F)** 2-chamber longitudinal strain (Ell__2Ch_), **(G)** 4-chamber longitudinal strain (Ell__4Ch_), and **(H)** radial strain (Rad). The **grey dotted line** represents the mean bias; the **pink dotted lines** the limits of agreement. The p values represent the difference in mean bias from zero bias using paired t-test. LAX = long-axis; LV = left ventricular; SAX = short-axis.

Overall, the Dice score between manual and automated segmentations was 0.93 ± 0.03% for the LV blood pool, 0.84 ± 0.02% for the LV myocardium, and 0.91 ± 0.03% for the RV blood pool segmentations. There was a good correlation between automatically and manually obtained cardiac volumes (LVEDV r = 0.99; LVESV r = 0.98; LVM r = 0.94; RVEDV r = 0.98; and RVESV r = 0.91), filling and ejection parameters (peak ejection rate r = 0.98; peak early filling rate r = 0.98; peak atrial filling rate r = 0.97 and AC r = 0.93) and strain (ε^circ^ r = 0.91; ε^rad^ r = 0.85; ε^long^ 2-chamber r = 0.91; and ε^long^ 4-chamber r = 0.89). The Bland-Altman plots for agreement between the pipeline and manual analysis are shown in Figures 1 and 2. There was no significant bias for cardiac volumes and filling and ejection parameters, except for RVESV (bias +1.80 ml; 2.3% of the mean RVESV; p = 0.01) and LVM (bias +2.95 ml; 2.7% of the mean LVM; p = 0.001). For strain, there was a significant bias for ε^circ^ (+0.75%; p < 0.001) and 2- and 4-chamber ε^long^ (+1.29%; p < 0.001 and +1.03%; p < 0.001, respectively). Lastly, there was no significant difference in mean absolute error between cardiac patients and healthy volunteers for the output parameters, except for LVESV (4.04 ± 4.04 ml vs. 6.65 ± 5.90 ml; p < 0.01) and AC (2.19 ± 2.17 ml vs. 3.30 ± 2.31 ml; p < 0.01) (Supplemental Table 2).

### Validation2

Table 1 shows the results of Validation2. For the total pipeline, sensitivity for volume parameters (volume curves, cardiac volume, and filling and ejection dynamics) was 94.99%, whereas the specificity was 82.93%. Stratified by group, the sensitivity was 94.83% in healthy subjects and 95.39% in cardiac patients. For strain assessment, sensitivity and specificity were 93.21% and 77.14%, respectively, and sensitivity for each subgroup was 92.69% in healthy subjects and 94.41% in cardiac patients. Supplemental Table 3 shows data for all the individual parameters. The total rate of CMRs flagged by the QCs was 26% in healthy volunteers and 32% in cardiac patients. The final rejection rate of the pipeline after clinician review was 15.2% for healthy subjects and 11% for the cardiac patients.

### Obtained Reference Values

A total of 2,029 subjects of the UK Biobank matched our criteria for healthy subjects and were processed using our pipe line (Supplemental Figure 2). During QC1, 222 cases (11%) were rejected for image quality. During QC2, 75 exams (4%) were automatically flagged for errors in cardiac volume output, whereas 119 (7%) were flagged for errors in strain analysis. Baseline characteristics of the remaining subjects are shown in Table 2. Reference values for cardiac volumes, cardiac function and filling and ejection parameters as well as ε^circ^, ε^long^ and ε^rad^ stratified by sex are shown in Tables 3 and 4. Supplemental Table 4 shows the regression analysis of changes in cardiac function in men and women with age.

## Discussion

In this study, we presented and validated a pipeline for automated analysis of ventricular function from cine CMR. Our pipeline is not solely a DL image analysis algorithm, but a framework that includes extensive QC steps to allow fully automatic processing of large numbers of CMR datasets without direct clinician oversight. We show that, using our proposed technique, we were able to obtain a detailed description of cardiac function in >2,000 healthy individuals. To the authors’ best knowledge, this is the first comprehensive framework for automated cine CMR analysis that approaches clinical standards of QC.

### Automated QC

QC is essential in developing DL algorithms for automated processing of clinical data, but has so far been mostly overlooked (12). In our framework, we implemented QC in 2 separate steps, a pre-analysis control of image quality, QC1, and a postanalysis control of the quality of the output parameters, QC2.

QC1 focused on detection of motion artefacts and off-axis planning of the obtained images. Motion artefacts do not result in static distortion of the image, which is easily recognized in post-analysis QC. Instead, the dynamic motion of the heart is affected due to incorporation of information from unrepresentative motion states (arrhythmias or mistriggering) or through- and in-plane motion (breathing artefacts). Similar to off-axis planning, these artefacts can have a significant impact on the computed parameters.

In QC2, we used a wide range of relevant criteria to evaluate the output of our pipeline, including clinical knowledge (similarity between LV and RV SV), anatomical relations (coverage of segmentations and images in LAX and SAX) and DL algorithms. This design ensured that erroneous and/or anomalous outputs were detected independent of their nature, even in cases not anticipated during development of the algorithms. This generalization facilitates implementation of the pipeline in clinical scenarios, such as large research databases or clinical practice, where the image quality and disease are not known a priori.

Techniques for automated QC have been previously proposed, such as motion artefact detection in brain magnetic resonance imaging (20), image quality evaluation in fetal (21) and cardiac (22) ultrasound, and detection of missing slices (23), off-axis planning (24), or segmentation errors (25) in CMR. So far, these techniques have been aimed at a single source of error and lack a generalized QC of the output based on clinical criteria. Robinson et al. (25) proposed a method to obtain segmentation quality scores for SAX segmentations from previous ratings in a large cohort of CMR segmentations. Obtaining quality scores from segmentations using this method, or other techniques that include uncertainty into segmentation networks, can complement our framework to further improve the quality of automated CMR analysis.

### Pipeline Validation

We validated the performance of the pipeline in 2 separate steps (Validation1 and Validation2). The direct comparison between automated and manual analysis in Validation1 demonstrated that the data obtained using our method was in high agreement for both segmentations (see Dice scores in Results subsection ‘Validation1’) as well as output (Figures 1 and 2). Only for LVM (+2.95 g), RVESV (+1.80 ml), and ε^circ^ ε^long^ strain (+0.75% and +1.03% to 1.29%, respectively) was there a small bias. However, these biases are within the range of inter- and intraobserver variabilities previously reported (6,26) and are unlikely to have significant clinical impact. The validation results for cardiac volumes (EDV, ESV, and SV) correspond well to the ones obtained in the original publication of the CNN_segment_ (10), showing its reproducibility. The Dice scores we obtained were slightly lower compared with the original publication of the segmentation network. The original network was trained and tested on segmentations made by the UK Biobank’s core analysis lab (6). In our paper, validation was performed against a new set of ground truth segmentations, performed by our own CMR cardiologists. The lower performance is therefore likely a reflection of the slight differences in training paradigms and segmentation strategies between cardiac CMR centers.

To investigate the detection of erroneous data by the QC steps of our image-processing pipeline, we evaluated its performance in a second, larger population. Manual analysis of all 700 cases in Validation2 is practically unrealistic. Therefore, we focused on critical review of the segmentations and output parameters to score their validity and evaluated the pipeline’s ability to detect the erroneous cases.

The results of Validation2 show that our 2-step QC robustly detects potential erroneous cases. Overall, the sensitivity of the pipeline to detect errors was high for both volume curves (94.99%) and strain (93.21%).

The specificity of the pipeline to correctly detect good cases was lower (82.93% for volume curves and 77.14% for strain). This is likely a consequence of the stringent QC criteria, resulting in flagging of cases with severely distorted anatomy (for example, after cardiac surgery) or abnormal volume curves (restricted ventricles with small volumes, low EF, and shallow early diastolic upslope of the curve). Although the lower specificity leads to unnecessary clinician review, we viewed it necessary to flag such cases to create a safe clinical workflow. However, the additional time for manual review is minimal because incorrectly flagged cases can be directly accepted upon review without adjustments.

It is noteworthy that, except for the lower specificity, our method performed similarly well in patients with cardiomyopathy as in healthy subjects, see the comparisons of absolute mean errors in validation1 (Supplemental Table 2) and sensitivity of error detection in validation2 (Table 1, Supplemental Table 3). Only for LVSV and AC were there small differences in mean absolute errors, but these are unlikely to have significant clinical impact. As can be appreciated from the Bland-Altman plots, the errors did not significantly increase at very high or very low values of the parameters. This further shows that the network has been robustly trained and is also accurate in outliers, such as patients with severe ventricular dilatation.

### Reference Values

After validation, we used our pipeline to obtain sex-specific reference values for the ventricular function parameters in a group of 2,029 healthy volunteers (Tables 3 and 4).

The values for cardiac volumes (EDV, ESV, SV, and LVM) obtained using our automated method are in correspondence with those manually obtained in previous sizable studies (6,7). In addition to these values, we also present reference values for filling and ejection dynamics and strain. The latter parameters have not previously been reported in large cohort studies. However, our results do correspond with the largest available study for filling and ejection parameters (27), and a meta-analysis of normal values for CMR-derived strain (28).

The total analysis time of the network was ~ 8 min/subject. This is significantly shorter than the time needed for manual or semiautomated segmentation and FT of the full cardiac cycle in SAX and LAX using the current state-of-the-art commercial software that requires frequent manual adjustments of semiautomated analysis in basal and apical slices of the acquisition.

### Study Limitations

At present, this method is designed using data from our Department of Cardiovascular Imaging and UK Biobank. Variability in type of CMR scanners and protocols results in variable image-characteristics between CMR labs. To obtain similar performance in other laboratories, additional training of the neural networks in the framework is needed using data from the new site. However, the principles, including the hardcoded QC measures, remain valid as vital components for automation of CMR analysis in general. If adapted using extra training input, this method can therefore potentially provide robust analysis in other large datasets, research studies, or even clinical CMR services. As part of the Open Science initiative, our method is available for further training and use via the corresponding author.

## Conclusions

We presented and validated a pipeline for automated analysis of cardiac function from cine CMR using DL. Our proposed framework includes comprehensive QC designed to detect potential erroneous results for clinician review, allowing fully autonomous processing of CMR exams. We showed that using this tool, we were able to obtain reference values in a large cohort (>2,000) of subjects to characterize cardiac function.

## Supplementary Material

Supplemental Video 2

Supplemental Video 1

Appendix

## Figures and Tables

**Central Illustration:**
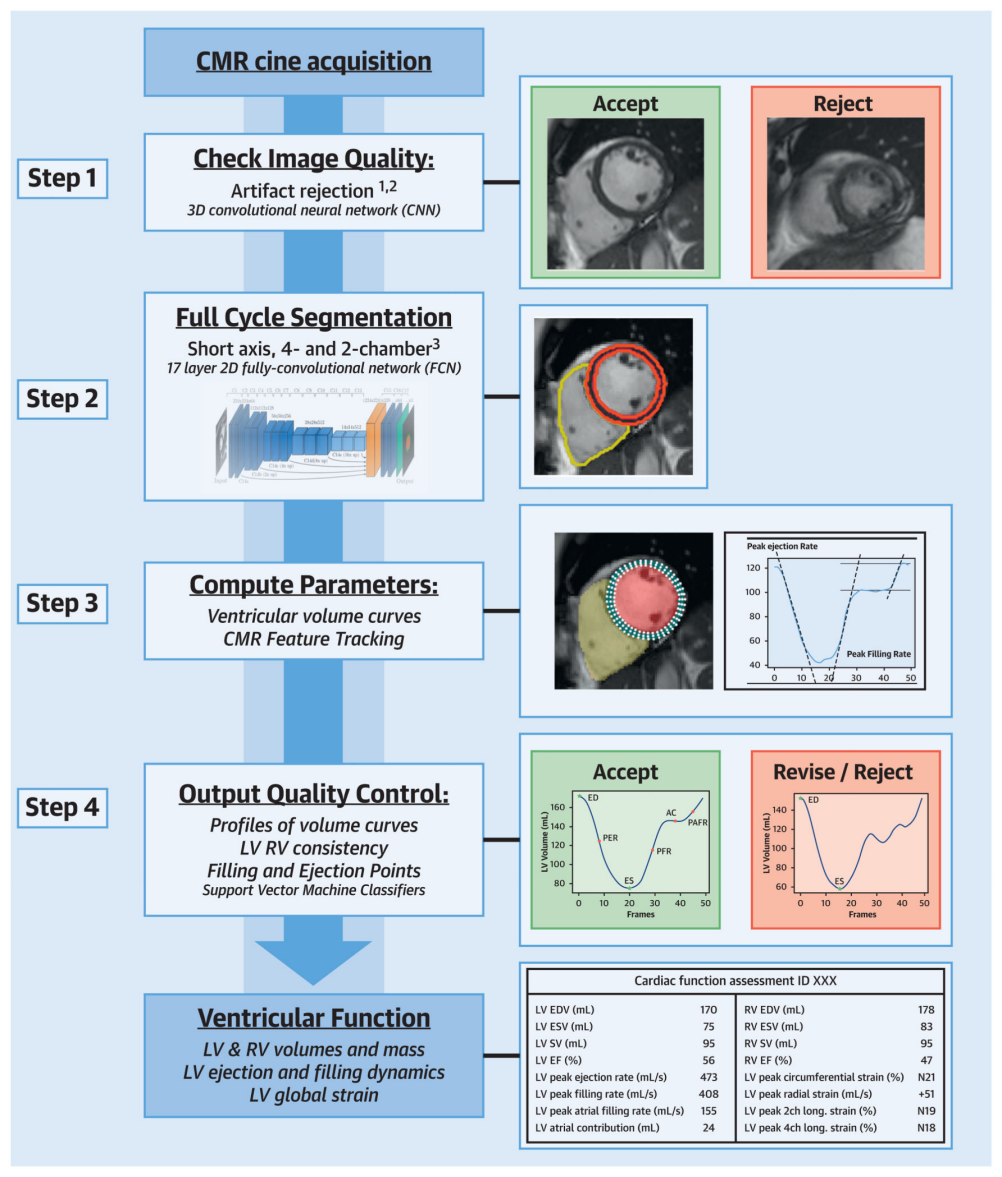
Total Image-Analysis Pipeline Including Pre- and Post-Analysis QC Steps An animation of the pipeline is shown in Supplemental Video 1. LV = left ventricle; QC = quality control; RV = right ventricle.

**Table 1:** Results of Validation2

	Validation Total Pipeline		

	Sensitivity, %	Specificity, %	BACC, %
Volumes			

Healthy subjects	94.83	86.57	90.70
Cardiac patients	95.39	76.78	86.09
Overall	94.99	82.93	88.96

Strains		

Healthy subjects	92.69	77.34	85.02
Cardiac patients	94.41	76.65	85.53
Overall	93.21	77.14	85.18

Sensitivity, specificity and balanced accuracy (BACC) of the pipeline in detecting inaccurate or unusual output versus correct output with respect to manual assessment are shown.

**Table 2:** Baseline Characteristics of the Healthy Subjects Included in the Analysis for Reference Values

	Age Groups, yrs		
	45–54 (n = 601)	55–64 (n = 706)	65–74 (n = 454)
Age, yrs	50 ± 2	59 ± 2	67 ± 2
Male	304 (50.58)	384 (54.39)	241 (53.08)
Systolic blood pressure, mm Hg	125 ± 11	130 ± 14	137 ± 15
Diastolic blood pressure, mm Hg	76 ± 7	77 ± 8	78 ± 8
Heart rate, beats/min	59 ± 8	60 ± 9	59 ± 8
Weight, kg	74 ± 11	73 ± 12	74 ± 10
Height, cm	171 ± 8	171 ± 12	172 ± 8
Body surface area, m^2^	1.87 ± 0.19	1.86 ± 0.18	1.87 ± 0.17
Body mass index, kg/m^2^	25.0 ± 2.7	24.8 ± 2.6	25.0 ± 2.6

Values are mean ± SD or n (%).

**Table 3:** Reference Values for Men by Automated Cine CMR Analysis

	Age Groups, yrs								
	45–54			55–64			65–74		
	Lower	Mean	Upper	Lower	Mean	Upper	Lower	Mean	Upper
Left ventricle

Volumes									
LV end-diastolic volume, ml	127	179	231	122	175	227	127	170	213
LV end-systolic volume, ml	48	77	106	46	73	99	51	72	93
LV stroke volume, ml	68	103	137	68	102	136	68	99	129
LV mass, g	71	104	137	73	100	126	74	98	123
Indexed LV end-diastolic volume, ml/m^2^	66	90	114	64	89	114	67	88	110
Indexed LV end-systolic volume, ml/m^2^	25	39	52	25	37	50	27	37	48
Indexed LV stroke volume, ml/m^2^	36	52	68	35	52	70	36	51	67
Indexed LV mass g/m^2^	38	52	66	39	51	63	40	51	61
LV ejection fraction, %	48	57	67	49	58	67	49	58	66
LV mass-to-volume ratio, g/ml	0.47	0.58	0.70	0.46	0.57	0.68	0.44	0.58	0.71
Filling and ejection dynamics									
Peak ejection rate, ml/s	362	502	643	343	483	623	329	466	604
Peak early filling rate, ml/s	239	417	594	202	369	537	167	332	496
Peak atrial filling rate, ml/s	77	254	431	102	269	436	63	222	382
Atrial contribution, ml	12	32	53	15	34	54	7	27	46
Atrial contribution, % of SV	10	32	54	18	34	50	6	28	50
Peak global strain									
Circumferential strain SAX, %	−14	−18	−26	−14	−19	−26	−15	−19	−25
TPK circumferential SAX, ms	280	341	423	279	339	420	291	340	408
Radial strain SAX, %	27	41	68	30	44	66	28	45	70
TPK radial SAX, ms	276	334	413	276	330	403	286	335	403
Longitudinal strain 2CH, %	−11	−16	−22	−11	−16	−22	−11	−16	−23
TPK longitudinal 2CH, ms	288	360	451	283	358	452	300	353	446
Longitudinal strain 4CH, %	−10	−15	−21	−9	−15	−21	−10	−16	−22
TPK longitudinal 4CH, ms	288	361	455	281	366	450	282	363	445

Right ventricle

RV end-diastolic volume, ml	132	196	259	128	188	247	139	188	237
RV end-systolic volume, ml	54	89	124	49	83	117	57	84	112
RV stroke volume, ml	72	105	139	69	105	140	73	104	135
Indexed RV end-diastolic volume, ml/m^2^	69	99	128	67	96	125	75	97	119
Indexed RV end-systolic volume, ml/m^2^	29	45	61	26	42	58	31	43	56
Indexed RV stroke volume, ml/m^2^	37	53	69	36	54	71	39	53	68
RV ejection fraction, %	46	54	62	47	56	65	46	55	64

Values are means and the lower and upper bound of the 95% prediction intervals. 2CH = 2-chamber; 4CH = 4-chamber; CMR = cardiac magnetic resonance; LAX = long-axis; LV = left ventricular; RV = right ventricular; SAX = short-axis; TPK = time to peak.

**Table 4:** Reference Values for Women by Automated Cine CMR Analysis

	Age Groups, yr								
	45–54			55–64			65–74		
	Lower	Mean	Upper	Lower	Mean	Upper	Lower	Mean	Upper
Left ventricle

Volumes
LV end-diastolic volume, ml	98	139	180	101	133	165	98	131	163
LV end-systolic volume, ml	34	55	76	33	51	70	34	50	66
LV stroke volume, ml	57	84	111	59	81	103	57	79	102
LV mass, g	51	71	91	52	69	87	54	70	86
Indexed LV end-diastolic volume, ml/m^2^	61	80	99	61	78	95	59	77	95
Indexed LV end-systolic volume, ml/m^2^	20	32	43	20	30	40	20	30	39
Indexed LV stroke volume, ml/m^2^	35	48	62	35	47	60	33	47	61
Indexed LV mass, g/m^2^	32	41	50	32	40	48	33	41	49
LV ejection fraction, %	51	61	70	52	61	71	52	61	70
LV mass-to-volume ratio, g/ml	0.42	0.51	0.61	0.42	0.52	0.62	0.43	0.54	0.65
Filling and ejection dynamics
Peak ejection rate, ml/s	266	386	507	263	370	477	259	363	466
Peak early filling rate, ml/s	231	364	497	207	322	436	179	302	425
Peak atrial filling rate, ml/s	54	204	355	73	223	373	82	234	386
Atrial contribution, ml	8	24	41	11	27	44	13	29	44
Atrial contribution, % of SV	13	29	44	17	33	50	20	36	51
Peak global strain
Circumferential strain SAX, %	−14	−20	−26	−14	−20	−26	−14	−20	−26
TPK circumferential SAX, ms	278	356	413	279	356	416	277	357	417
Radial strain SAX, %	24	47	68	28	47	69	27	46	68
TPK radial SAX, ms	275	358	401	275	358	407	271	360	408
Longitudinal strain 2CH, %	−11	−17	−22	−10	−17	−22	−11	−17	−21
TPK longitudinal 2CH, ms	277	374	451	281	374	451	277	378	458
Longitudinal strain 4CH, %	−9	−15	−21	−10	−15	−21	−10	−16	−21
TPK longitudinal 4CH, ms	274	372	451	278	372	453	284	378	453

Right ventricle

RV end-diastolic volume, ml	97	142	188	101	139	176	99	132	164
RV end-systolic volume, ml	34	58	82	33	56	79	34	52	70
RV stroke volume, ml	57	84	111	60	83	106	60	80	99
Indexed RV end-diastolic volume, ml/m^2^	59	82	105	62	80	99	58	78	98
Indexed RV end-systolic volume, ml/m^2^	21	33	46	20	33	45	20	31	41
Indexed RV stroke volume, ml/m^2^	34	48	62	36	48	60	34	48	62
RV ejection fraction, %	50	59	68	50	60	70	54	61	68

Abbreviations as in Table 3.

## Perspectives

**Competency in Medical Knowledge:** CMR can provide sensitive biomarkers for cardiac function. However, analysis is time and labor intensive. DL can automate CMR analysis, but adequate QC of the algorithms is pivotal.

**Translational Outlook 1:** This is the first study to develop a comprehensive method for DL-based CMR analysis that includes extensive QC measures to flag potential erroneous results.

**Translational Outlook 2:** We show that our pipeline enables detailed characterization of cardiac function in an automated and accurate way in large-scale studies and potentially clinical practice.

## Abbreviations and Acronyms

ε: myocardial strain
AC: atrial contribution
CMR: cardiac magnetic resonance
CNN: convolutional neural network
DL: deep learning
EDV: end-diastolic volume
EF: ejection fraction
ESV: end-systolic volume
FT: feature tracking
LAX: long-axis
LV: left ventricle/ventricular
LVM: left ventricular mass
QC: quality control
RV: right ventricle/ventricular
SAX: short-axis
SV: stroke volume
SVM: support vector machine
