# Supplementary material for: Fully Automated, Quality-Controlled Cardiac Analysis From CMR: Validation and Large-Scale Application to Characterize Cardiac Function
Source: JACC Cardiovasc Imaging. Author manuscript; Available in PMC 2020 Mar 7. (PMC7060799; doi:10.1016/j.jcmg.2019.05.030)
Supplement: Appendix [file EMS84281-supplement-Appendix.docx]

**Supplementary Figure 1. Case-review panel for *Validation2*.** The display for manual review of the output. Reviewed are LV volume curve (left graph), LV strain curves (middle graph) and RV volume curve (right graph), shown simultaneously with a video of the segmentations per slice. An animation of the case-review panel is shown in Video 2.

**Supplementary Figure 2: Flowchart of selection for reference values.**

**Supplementary Table 1. Exclusion criteria used for the selection of healthy volunteers from the UK Biobank database.**

| **Exclusion criteria** | | **Number of cases excluded (%)** |
| --- | --- | --- |
| **Age** |  |  |
|  | >75 years | 1 (0%) |
| **Medical conditions** | |  |
|  | Hypertension | 2705 (28%) |
|  | High cholesterol | 1740 (18%) |
|  | Asthma | 1287 (13%) |
|  | Hypothyroidism / myxoedema | 587 (6%) |
|  | Diabetes | 406 (4%) |
|  | Essential hypertension | 431 (4%) |
|  | Angina | 251 (3%) |
|  | Heart attack/myocardial infarction | 203 (2%) |
|  | Type 2 diabetes | 186 (2%) |
|  | Atrial fibrillation | 151 (2%) |
|  | Miscarriage | 151 (2%) |
|  | Iron deficiency anaemia | 148 (2%) |
|  | Stroke | 130 (1%) |
|  | Emphysema/chronic bronchitis | 126 (1%) |
|  | Hyperthyroidism/thyrotoxicosis | 92 (1%) |
|  | Heart valve problem/heart murmur | 91 (1%) |
|  | Transient ischaemic attack | 91 (1%) |
|  | Chronic obstructive airways disease | 80 (1%) |
|  | Pulmonary embolism +/- DVT | 70 (1%) |
|  | Ulcerative colitis | 68 (1%) |
|  | Heart arrhythmia | 63 (1%) |
|  | Sleep apnoea | 54 (1%) |
|  | Polymyalgia rheumatica | 53 (1%) |
|  | Irregular heart beat | 84 (1%) |
|  | Gestational hypertension/pre-eclampsia | 65 (1%) |
|  | Diabetic eye disease | 44 (0%) |
|  | Doctor diagnosed bronchiectasis | 43 (0%) |
|  | Heart/cardiac problem | 39 (0%) |
|  | Anaemia | 38 (0%) |
|  | Bronchiectasis | 36 (0%) |
|  | Pernicious anaemia | 34 (0%) |
|  | Rheumatic fever | 33 (0%) |
|  | Ankylosing spondylitis | 29 (0%) |
|  | Sarcoidosis | 27 (0%) |
|  | Crohn’s disease | 24 (0%) |
|  | Peripheral vascular disease | 23 (0%) |
|  | Supraventricular tachycardia | 23 (0%) |
|  | Gestational diabetes | 22 (0%) |
|  | Sjogren’s syndrome/sicca syndrome | 20 (0%) |
|  | Type 1 diabetes | 18 (0%) |
|  | Low platelets/platelet disorder | 17 (0%) |
|  | Clotting disorder/excessive bleeding | 15 (0%) |
|  | Other respiratory problems | 15 (0%) |
|  | Retinal artery/vein occlusion | 15 (0%) |
|  | Systemic lupus erythematosis | 13 (0%) |
|  | Haemochromatosis | 12 (0%) |
|  | Renal failure not requiring dialysis | 12 (0%) |
|  | Gestational diabetes | 11 (0%) |
|  | Pericarditis | 11 (0%) |
|  | Atrial flutter | 11 (0%) |
|  | Glomerulnephritis | 11 (0%) |
|  | Renal/kidney failure | 10 (0%) |
|  | Grave’s disease | 10 (0%) |
|  | Nephritis | 9 (0%) |
|  | Mitral regurgitation/incompetence | 9 (0%) |
|  | Anorexia/bulimia/other eating disorder | 9 (0%) |
|  | Heart failure/pulmonary edema | 8 (0%) |
|  | Connective tissue disorder | 8 (0%) |
|  | Hereditary/genetic haematological disorder | 7 (0%) |
|  | Adrenocortical insufficiency/Addison’s disease | 7 (0%) |
|  | Lymphoedema | 7 (0%) |
|  | Kidney nephropathy | 7 (0%) |
|  | Leg claudication/intermittent claudication | 7 (0%) |
|  | Myocarditis | 6 (0%) |
|  | Cardiomyopathy | 6 (0%) |
|  | Aortic stenosis | 6 (0%) |
|  | Inflammatory bowel disease | 6 (0%) |
|  | Surgery/amputation of toe | 6 (0%) |
|  | Hypertrophic cardiomyopathy | 6 (0%) |
|  | Hypopituitarism | 6 (0%) |
|  | Polycythaemia vera | 5 (0%) |
|  | Aortic regurgitation/incompetence | 5 (0%) |
|  | Neutropenia/lymphopenia | 5 (0%) |
|  | Monoclonal gammopathy/not myeloma | 5 (0%) |
|  | Mitral valve disease | 5 (0%) |
|  | Vasculitis | 5 (0%) |
|  | Mitral valve prolapse | 4 (0%) |
|  | Aortic aneurysm | 4 (0%) |
|  | Interstitial lung disease | 4 (0%) |
|  | Hyperprolactinaemia | 4 (0%) |
|  | Surgery/amputation of leg below the knee | 4 (0%) |
|  | Emphysema | 4 (0%) |
|  | Liver failure/cirrhosis | 3 (0%) |
|  | Aplastic anaemia | 3 (0%) |
|  | Antiphospholipid syndrome | 3 (0%) |
|  | Diabetic neuropathy/ulcers | 3 (0%) |
|  | Pleural plaques (not known asbestosis) | 3 (0%) |
|  | Haemophilia | 3 (0%) |
|  | Wegners granulmatosis | 3 (0%) |
|  | Wolff-Parkinson-White syndrome | 3 (0%) |
|  | Giant cell/temporal arteritis | 3 (0%) |
|  | Myositis/myopathy | 2 (0%) |
|  | Fibrosing alveolitis/unspecified alveolitis | 2 (0%) |
|  | Sickle cell disease | 1 (0%) |
|  | Microscopic polyarteritis | 1 (0%) |
|  | Hyperaldosteronism/Conn’s syndrome | 1 (0%) |
|  | Polymyositis | 1 (0%) |
|  | Alcoholic liver disease/alcoholic cirrhosis | 1 (0%) |
|  | Diabetes insipidus | 1 (0%) |
|  | Pericardial problem | 0 (0%) |
|  | IgA nephropathy | 2 (0%) |
|  | Pleural effusion | 2 (0%) |
|  | Respiratory failure | 1 (0%) |
|  | Sick sinus syndrome | 1 (0%) |
|  | Myeloproliferative disorder | 1 (0%) |
|  | Surgery/amputation of leg above the knee | 1 (0%) |
|  | Pericardial effusion | 0 (0%) |
| **Medication** | |  |
|  | Cholesterol lowering medication | 2208 (23%) |
|  | Blood pressure medication | 2266 (24%) |
|  | Hormone replacement therapy | 594 (6%) |
|  | Insulin | 81 (1%) |
| **Symptoms** | |  |
|  | Shortness of breath walking on level ground | 977 (10%) |
|  | Chest pain due to walking ceases when standing still | 529 (6%) |
|  | Chest pain when walking uphill or hurrying | 464 (5%) |
|  | Unable to walk up hills or to hurry | 254 (3%) |
| **Smoking history** | |  |
|  | Ex-smoker | 3118 (32%) |
|  | Current smoker | 823 (8%) |
| **Habitus** | |  |
|  | BMI ≥30 | 1820 (19%) |
| **Ethnicity** | |  |
|  | Indian | 69 (1%) |
|  | Pakistani | 24 (0%) |
|  | Caribbean | 32 (0%) |
|  | Chinese | 27 (0%) |
|  | African | 22 (0%) |
|  | Any other mixed background | 19 (0%) |
|  | Prefer not to answer | 16 (0%) |
|  | Any other Asian background | 14 (0%) |
|  | White and Black Caribbean | 8 (0%) |
|  | White and Black African | 8 (0%) |
|  | White and Asian | 7 (0%) |
|  | Bangladeshi | 3 (0%) |
|  | Do not know | 2 (0%) |
|  | Asian or Asian British | 1 (0%) |
|  | Any other Black background | 0 (0%) |
|  | Black or Black British | 0 (0%) |
|  | Other ethnic group | 39 (0%) |

**Supplementary Table 2. Mean absolute errors in ventricular function parameters using the automated pipeline vs. manual assessment.** Level of significance denotes the difference in absolute error between healthy subjects and cardiac patients. **** *p*-value < 0.0001; *** *p*-value < 0.001; ** *p*-value < 0.01; * *p*-value < 0.05.

|  |  | ***Mean absolute error of automated analysis compared to manual*** | | | |
| --- | --- | --- | --- | --- | --- |
|  |  | **Overall** | **Healthy Subjects** | **Cardiac patients** | *Level of significance* |
| **Left Ventricle** | |  |  |  |  |
| *Volumes* | |  |  |  |  |
|  | LVEDV (mL) | 5.81±3.31 | 5.40±2.80 | 6.22±3.72 |  |
|  | LVESV (mL) | 5.52±3.73 | 5.00±3.21 | 6.05±4.11 |  |
|  | LVSV (mL) | 5.35±5.22 | 4.04±4.04 | 6.65±5.90 | * |
|  | LVM (g) | 9.13±6.47 | 8.80±6.52 | 9.45±6.40 |  |
|  | iLVEDV (mL/m^2^) | 3.09±1.70 | 2.99±1.58 | 3.19±1.81 |  |
|  | iLVESV (mL/m^2^) | 2.93±1.91 | 2.75±1.76 | 3.11±2.03 |  |
|  | iLVSV (mL/m^2^) | 2.80±2.68 | 2.20±2.21 | 3.39±2.97 | * |
|  | iLV mass (g/m^2^) | 4.88±3.42 | 4.89±3.63 | 4.87±3.20 |  |
|  | LVEF (%) | 3.45±2.53 | 3.08±2.17 | 3.81±2.80 |  |
|  | LV mass-to-volume ratio | 0.07±0.06 | 0.07±0.06 | 0.07±0.05 |  |
| *Filling and ejection dynamics* | |  |  |  |  |
|  | PER (mL/s) | 17.10±12.27 | 19.36±15.92 | 14.83±6.11 |  |
|  | PFR (mL/s) | 19.08±14.37 | 21.59±17.29 | 16.58±10.07 |  |
|  | PAFR (mL/s) | 16.34±10.67 | 15.19±10.88 | 17.49±10.32 |  |
|  | AC (mL) | 2.74±2.31 | 2.19±2.17 | 3.30±2.31 | * |
| *Peak global strain* | |  |  |  |  |
|  | Circumferential SAX (%) | 1.47±0.97 | 1.39±0.91 | 1.54±1.01 |  |
|  | TPK Circumferential SAX (msec) | 23.18±19.31 | 17.70±15.43 | 28.66±21.15 |  |
|  | Radial Strain SAX (%) | 4.36±3.08 | 4.35±2.86 | 4.38±3.29 |  |
|  | TPK Radial SAX (msec) | 26.65±23.22 | 21.64±21.23 | 31.66±24.03 |  |
|  | Longitudinal 2CH LAX (%) | 1.59±1.08 | 1.74±1.17 | 1.43±0.95 |  |
|  | TPK longitudinal 2CH LAX (msec) | 23.63±19.53 | 20.74±19.85 | 26.52±18.76 |  |
|  | Longitudinal 4CH LAX (%) | 1.83±1.19 | 1.86±1.16 | 1.81±1.22 |  |
|  | TPK longitudinal 4CH LAX (msec) | 23.38±17.87 | 21.66±16.87 | 25.10±18.65 |  |
| **Right Ventricle** | |  |  |  |  |
|  | RVEDV (mL) | 6.71±2.90 | 6.53±2.99 | 6.88±2.80 |  |
|  | RVESV (mL) | 6.68±3.26 | 6.28±3.69 | 7.07±2.73 |  |
|  | RVSV (mL) | 8.51±5.96 | 9.00±5.62 | 8.02±6.24 |  |
|  | iRVEDV (mL/m^2^) | 3.60±1.63 | 3.62±1.76 | 3.59±1.48 |  |
|  | iRVESV (ml/m^2^) | 3.56±1.75 | 3.46±2.06 | 3.66±1.37 |  |
|  | iRVSV (ml/m^2^) | 4.55±3.15 | 4.97±3.06 | 4.13±3.18 |  |
|  | RVEF (%) | 4.99±3.45 | 5.34±3.48 | 4.63±3.38 |  |

**Supplementary Table 3. Sensitivity, specificity and balanced accuracy (BACC) for volume and strain parameters of the individual output parameters during *Validation2*.** PER; peak ejection rate, PEFR; peak early filling rate, PAFR; peak atrial filling rate, AC; atrial contribution, ε^circ^; peak global circumferential strain, ε^rad^; peak global radial strain, ε^long 2ch^; peak longitudinal strain in 2-chamber view, ε^long 4ch^; peak longitudinal strain in 4-chamber view.

|  |  | **Validation total pipeline** | | |
| --- | --- | --- | --- | --- |
|  |  | SENSITIVITY (%) | SPECIFICITY (%) | BACC (%) |
| **PER** | *Healthy Subjects* | 95.12 | 85.26 | 90.19 |
|  | *Cardiac Patient* | 95.83 | 76.97 | 86.40 |
|  | ***Overall*** | **95.32** | **83.93** | **88.96** |
| **PEFR** | *Healthy Subjects* | 94.40 | 84.53 | 89.47 |
|  | *Cardiac Patient* | 95.92 | 76.16 | 86.04 |
|  | ***Overall*** | **94.83** | **82.13** | **88.48** |
| **PAFR** | *Healthy Subjects* | 95.38 | 85.95 | 90.67 |
|  | *Cardiac Patient* | 94.23 | 77.70 | 85.97 |
|  | ***Overall*** | **95.05** | **83.59** | **89.32** |
| **AC** | *Healthy Subjects* | 95.14 | 84.89 | 93.68 |
|  | *Cardiac Patient* | 94.83 | 77.03 | 85.44 |
|  | ***Overall*** | **95.05** | **82.62** | **88.99** |
| **ε^circ^** | *Healthy Subjects* | 92.80 | 79.20 | 86.00 |
|  | *Cardiac Patient* | 97.55 | 81.08 | 89.31 |
|  | ***Overall*** | **94.24** | **79.63** | **86.93** |
| **ε^rad^** | *Healthy Subjects* | 92.80 | 79.20 | 86.00 |
|  | *Cardiac Patient* | 94.84 | 77.78 | 86.31 |
|  | ***Overall*** | **93.40** | **78.82** | **86.11** |
| **ε^long 2Ch^** | *Healthy Subjects* | 91.43 | 73.33 | 82.38 |
|  | *Cardiac Patient* | 92.36 | 74.42 | 83.39 |
|  | ***Overall*** | **91.72** | **73.58** | **82.65** |
| **ε^long 4Ch^** | *Healthy Subjects* | 93.75 | 77.61 | 85.68 |
|  | *Cardiac Patient* | 92.90 | 73.33 | 83.12 |
|  | ***Overall*** | **93.50** | **76.54** | **85.02** |

**Supplementary Table 4. Linear regression coefficients for cardiac function parameters and age in men and women.** ^a^ and ^b^ denotes beta and Pearson’s r obtained from linear regression of parameter versus age for males and females, respectively. **** *p*-value < 0.0001; *** *p*-value < 0.001; ** *p*-value < 0.01; * *p*-value < 0.05. LV; left ventricle, TPK; time to peak, RV; right ventricle.

|  |  | **Males** | | |  | **Females** | | |
| --- | --- | --- | --- | --- | --- | --- | --- | --- |
|  |  | Beta^a^ | Pearson's r^b^ | Level of significance^b^ |  | Beta^a^ | Pearson's r^b^ | Level of significance^b^ |
| **Left Ventricle** | |  |  |  |  |  |  |  |
| *Volumes* | |  |  |  |  |  |  |  |
|  | LV end-diastolic volume (mL) | -1.04 | -0.25 | **** |  | -0.36 | -0.13 | ** |
|  | LV end-systolic volume (mL) | -0.56 | -0.25 | **** |  | -0.21 | -0.14 | ** |
|  | LV stroke volume (mL) | -0.50 | -0.20 | **** |  | -0.15 | -0.09 |  |
|  | LV mass (g) | -0.50 | -0.22 | **** |  | 0.00 | 0.00 |  |
|  | indexed LV end-diastolic volume (mL/m^2^) | -0.39 | -0.20 | **** |  | -0.12 | -0.09 |  |
|  | indexed LV end-systolic volume (mL/m^2^) | -0.22 | -0.21 | **** |  | -0.09 | -0.11 | * |
|  | indexed LV stroke volume (mL/m^2^) | -0.17 | -0.14 | **** |  | -0.03 | -0.03 |  |
|  | indexed LV mass (g/m^2^) | -0.17 | -0.17 | **** |  | -0.04 | 0.06 |  |
|  | LV ejection fraction (%) | 0.07 | 0.10 | * |  | 0.05 | 0.07 |  |
| *Filling and ejection dynamics* | |  |  |  |  |  |  |  |
|  | Peak ejection rate (mL/s) | -2.77 | -0.25 | **** |  | -0.91 | -0.11 | * |
|  | Peak early filling rate (mL/s) | -5.51 | -0.42 | **** |  | -3.68 | -0.37 | **** |
|  | Peak atrial filling rate (mL/s) | 1.19 | 0.10 | ** |  | 2.14 | 0.19 | **** |
|  | Atrial contribution (mL) | 0.20 | 0.12 | *** |  | 0.20 | 0.15 | *** |
|  | Atrial contribution (% of SV) | 0.30 | 0.08 | * |  | 0.00 | -0.08 |  |
| *Peak global strain* | |  |  |  |  |  |  |  |
|  | Circumferential strain SAX (%) | -0.06 | -0.15 | **** |  | -0.07 | -0.18 | *** |
|  | TPK Circumferential SAX (msec) | 0.32 | 0.07 |  |  | 0.25 | 0.05 |  |
|  | Radial Strain SAX (%) | 0.11 | 0.09 | * |  | 0.16 | 0.10 | * |
|  | TPK Radial SAX (msec) | 0.23 | 0.05 |  |  | 0.31 | 0.07 |  |
|  | Longitudinal strain 2CH (%) | 0.03 | 0.04 |  |  | 0.02 | 0.02 |  |
|  | TPK longitudinal 2CH (msec) | 0.14 | 0.02 |  |  | 0.25 | 0.05 |  |
|  | Longitudinal strain 4CH (%) | -0.02 | -0.03 |  |  | 0.04 | 0.04 |  |
|  | TPK longitudinal 4CH (msec) | -0.57 | -0.10 | * |  | -0.08 | -0.01 |  |
| **Right Ventricle** | |  |  |  |  |  |  |  |
|  | RV end-diastolic volume (mL) | -0.94 | -0.22 | *** |  | -0.41 | -0.14 |  |
|  | RV end-systolic volume (mL) | -0.60 | -0.24 | **** |  | -0.25 | -0.14 |  |
|  | RV stroke volume (mL) | -0.34 | -0.14 | * |  | -0.16 | -0.09 |  |
|  | indexed RV end-diastolic volume (mL/m^2^) | -0.40 | -0.20 | *** |  | -0.12 | -0.08 |  |
|  | indexed RV end-systolic volume (ml/m^2^) | -0.27 | -0.22 | ** |  | -0.10 | -0.11 |  |
|  | indexed RV stroke volume (ml/m^2^) | -0.13 | -0.11 |  |  | -0.02 | -0.02 |  |
|  | RV ejection fraction (%) | 0.08 | 0.13 |  |  | 0.06 | 0.09 |  |
